# Book Review

**Published:** 1920-09

**Authors:** 


**John Coakley Lettsom and the Foundation of the Medical Society.** By Sir St. Clair Thomson. Pp. 63. Illustrated. London: Harrison & Sons. 1918. Price, 2s. 6d.—Lettsom, one of the founders of the Medical Society, died in 1805, and though one might expect an array of interesting facts would be available for the historian, it is given to few to make one feel in touch with a personality, and to call up an almost living picture of his life and activities. In this, however, Sir St. Clair Thomson has been singularly successful, and his brochure and his descriptions of the various phases and episodes of the life of this distinguished eighteenth-century physician cannot fail to interest the reader. One of the questions of the day in 1800 was the price of bread, and one gathers from Lettsom's writings that the price of bread in 1774 was 8d., but that by 1800 " we find him writing, ' At present our quartern loaf is 1s. 6d., a sum truly oppressive to the poor.' " The author continues, " With our present-day quartern loaf at only 9d., after three years of world war, our population may take courage by seeing what our forefathers had to endure in the Napoleonic struggles." Lettsom in his " Hints respecting a substitute for Wheat Bread " recommends Indian corn flour, and also refers to the merits of maize flour and of potatoes as other partial substitutes. How history repeats itself ! But it is the references to Lettsom's residences, his favourite holiday, his American friendships, associations with celebrated people of his day, *e.g.* Dr. Samuel Johnson and Boswell, his habits of life, his methods of work, and his character that constitute the essential interest of this disquisition.

**The Nation's Welfare.** By Major-General Sir BERTRAND DAWSON, G.C.V.O. London: Cassell & Co. Ltd. 1918. Pp. 40. Price 6s.—This reprint of Sir Bertrand Dawson's Cavendish Lectures embodies his ideas on the organisation of National Medical Service and the Ministry of Health. The lectures were published in the *British Medical Journal,* and should be familiar to all medical men who are alive to the important problems now before the profession in the country. Some of Sir Bertrand Dawson's suggestions have already been embodied in the constitution of the Ministry of Health, notably the Advisory Council of Medical Men, of which he is now Chairman. Many of his other views seem likely to be brought to practical fruition sooner or later ; and this pamphlet has therefore a great claim on the attention of all members of our profession. Very important is the insistence of the necessity in any national scheme for leaving all technical questions entirely in the hands of doctors, who alone have the right to decide them. Of almost equal importance is the view herein expressed that in any organised State Medical Service practitioners should be free agents not whole-time officials, and that their needs, both material (such as salaries, etc.) and educational (study leave, postgraduate work, research), should be provided for adequately by the State or by local authorities. We have picked out only two salient points, but the pamphlet teems with important ideas and suggestions, and should be carefully read and digested by all medical men, even the oldest.

**" The Journal of Industrial Hygiene."**—Vol. I., Nos. 1 to 4. Edited by DAVID L. EDSALL, M.D., S.D. (United States), and A. F. STANLEY KENT, A.M., D.Sc. (Great Britain). Monthly. London: The Macmillan Co. Price 21s. per annum.—We are pleased to welcome this excellent periodical, which is, we think, destined to take a large share in the development of this important subject. Its appeal is wide. In the first four numbers there are exhaustive articles on such varied subjects as

"The Problem of Fatigue," "Factory Inspection," "The Study of the Dust Content of Air," and "Poisoning by Compounds of the Aromatic Series." Then there are numerous articles on medical and surgical subjects and abstracts of current literature. These titles indicate the wide scope which the Editors have given to their *Journal*, with the result that all workers on subjects connected in any way with the health of the nation will find therein matter to interest and inform them. It is not possible in a brief review to deal with the separate articles in detail; they are all written by contributors with practical experience of their subjects, and are remarkable for clearness, conciseness and accuracy. Industrial chemistry and toxicology is well represented by the articles on "Manganese Poisoning" by the American Editor and others, the "Poisoning by Benzene Compounds," by Alice Hamilton, a subject recently brought into prominence by cases of TNT poisoning; "Lead Poisoning," by the same author, which though dealing with the industrial questions involved mainly from the American standpoint, has some very interesting paragraphs on the pathology of the condition; and again one on other inorganic poisons, which includes sections of much value on carbon monoxide, arsenic and phosphorus, as well as certain metals. Other subjects are equally well treated; and there is an individuality about the various articles which makes them very pleasant reading. The *Journal* is clearly and well printed on excellent paper, and the charts, diagrams and illustrations are finely reproduced. Good bibliographies dealing with recent papers are appended to a large number of articles, and will be found most useful.

We congratulate the Editors on the first four numbers, and wish the *Journal* all the success which its many excellencies deserve.

**Psychoanalysis and its Place in Life.** By M. K. BRADBY. Pp. xi., 266. London: Oxford Medical Publications. 1919. Price 8s. 6d. net.—Miss Bradby's book is written from a point of view other than medical, and affords a happy addition to the more conventional works on psychoanalysis to which we are accustomed. It should, moreover, help to dispel prejudices with regard to the subject which some of its advocates have aroused.

The earlier chapters contain an excellent résumé of a number of psychoanalytic theories. While the authoress pays a full tribute to the value of the pioneer work of the Freundian school, she indicates at the same time a broader horizon to the student of psychoanalysis. Art, ethics, morals and education she brings within the sphere of psychoanalytic teaching and research.

Miss Bradby treats her theme in a highly subjective manner, and in the opinion of some, at any rate. adds interest by the prominence given to her personal point of view. Thereby she lays herself open to criticism by those who maintain that psychology loses in scientific value when it departs from objective considerations of thought and conduction. Strictly, it may be no part of pure psychology to consider moral and ethical values as such, but the patient does not allow the clinician to adopt this entirely impersonal attitude. To the neurotic the subjective treatment of his case is of great moment. Herein lies the greater clinical value of Jung's method, to those who practice psychotherapy, as compared with the more strictly scientific method of Freund. It is, then, this unscientific blend in Miss Bradby's book which helps to render it attractive to the clinician, while the authoress herself finds the greatest interest in the social and educational aspects of her work.

**Half a Century of Smallpox and Vaccination** (The Milroy Lectures, 1919). By JOHN C. McVAIL, M.D., LL.D. Edinburgh: E. & S. Livingstone. (N.D.) Price 5s. 6d. net.—The publication of this survey is opportune in view of the facts disclosed in the final Report of the Local Government Board, that in 1917 only 43.3 per cent. of children were "successfully vaccinated," compared with 61 per cent. in 1898, while in the same period the "exemptions" have risen from 5 per cent. to 37.9; and that while, owing to war conditions, the adult male population is better protected by re-vaccination than formerly, only about half the children under 10 years of age have been vaccinated, so that the measures for repression which have hitherto proved successful cannot be implicitly relied upon in a population so largely unprotected. Under these circumstances a study of Dr. McVail's well-informed and judicious survey cannot fail to be of service to every member of the medical profession.

**War Diseases and Pensions.** By R. M. WILSON, M.B., and W. M. T. WILSON, M.B. Pp. 71. London: Oxford Medical Publications. 1919. Price 3s. 6d.—This little book has two purposes. The purpose avowed in its title is to give to the medical profession the guidance which it sorely needs in decision upon war pensions. But for this purpose the book is unfortunately quite inadequate. It does good service in calling attention to the fact that no case has been diagnosed adequately until the causation has been determined. Particularly is this true of "D.A.H.," to which at least half the book is devoted. This indeed appears to be the main though unavowed purpose of the book, to propagate certain ideas as to the causation of "D.A.H." The theory held by the authors is that "D.A.H." is not a disease but a symptom, and that it is always a symptom of some infection or toxæmia. With the first part of this theory we cordially agree. As to the second, we are convinced the writers do not realise the facts of warfare, or they would attribute more importance to the psychic factor in "D.A.H."

**The Soldier's First Aid.** By R. C. Wood, Q.M.S., C.A.M.C. Pp. 93. Toronto: The Macmillan Company of Canada. 1917. Price 2s. 6d. net.—An admirable little handbook, full of terse, accurate directions. The illustrations are first-rate, the language is simple. This is one of the best books for teaching first-aid and ambulance work that we have seen.

**Diabetes and its Dietetic Treatment.** By M. D. Basu, Major, I.M.S. (retired). Tenth Edition. Pp. 89. Allahabad: The Panini Office. 1919. Price 1 rupee, 8 annas.—The editions of Major Basu's book appear with great rapidity, bespeaking no doubt its popularity in India. The principal additions in this tenth edition consist of further insistence on the importance of vitamines in any diabetic diet, and a definite statement in the preface that in diabetes "the sugar in the blood plays the part of an antitoxin," an opinion, we venture to think, based on insufficient scientific evidence.

**Syphilis in Childhood.** By Leonard Findlay, M.D. Pp. xii., 154. London: Oxford Medical Publications. 1919. Price 8s. 6d. net.—In these days of Infant Welfare Centres and Antenatal Clinics this little handbook will prove of great practical value. Syphilis masquerades in countless disguises, as though doing its best to escape detection, and it behoves every medical man to recognise its presence whatever form it may assume.

Dr. Findlay describes very clearly the principal manifestations of the disease in childhood and their differential diagnosis. He very rightly insists on the value of a Wassermann reaction in clearing up many a doubtful diagnosis, and the method he employs for obtaining sufficient blood for the test even from a tiny infant deserves special notice and is well illustrated.

The luetin test he finds most unreliable in the case of children. Perhaps the most generally useful chapter in the book is that which deals with treatment.

The dosage of the different salvarsan derivatives is clearly stated, and the methods of administering these drugs intravenously and intramuscularly to children are given in detail.

Dr. Findlay's observations on the results of the treatment of expectant mothers are particularly encouraging and deserve to be widely known.

The volume is concisely written and well illustrated.

It will prove a useful addition to any doctor's professional library.

